# Cases of an Eruptive Disease Arising from the Use of Cubebs

**Published:** 1832-03

**Authors:** John North


### ERUPTIVE DISEASE FROM THE USE OF CUBEBS.

### *Cases of an Eruptive Disease arising from the Use of Cubebs.* By JOHN NORTH, F.L.S.

THE reputation of a medical practitioner in the estimation of the public as frequently depends upon the knowledge he possesses of trifling facts that are connected with his profession, as upon his intimate acquaintance with the nature and treatment of the most formidable maladies. A circumstance recently fell under my observation which illustrates the truth of this assertion: Two footmen, in a family of distinction, were suddenly attacked with a severe eruptive disease, resembling Urticaria, but differing in some respects from that complaint. The symptoms of general disturbance ran rather high, and the family became alarmed: their usual medical attendant was consulted, and, with more sincerity than judgment, he confessed that "he could not possibly conceive what the disease was, or from what cause it had arisen." Another practitioner happened to be in attendance upon one of the members of the family, and his attention was attracted by the appearance of the men servants: he had several times seen such an eruptive disease produced by the use of cubebs, and, upon being informed that a mystery hung over the nature and cause of the complaint, he at once suggested that cubebs had been taken, and that the local and general symptoms would soon disappear. The fact was precisely as he supposed. The men had had gonorrhœa; had dosed themselves freely with cubebs, and from the use of this medicine the eruptive disease had arisen. They were placed under the care of the surgeon who had shewn a knowledge of the nature of their complaint, and under the use of mild aperients, with a few days' confinement to the house, they were entirely relieved. The confidence of the family in their former medical attendant was destroyed, and the second practitioner has subsequently retained their friendship.

This anedote is mentioned as an apology for relating the following cases, which happened in my own practice. I am aware that to many surgeons the fact is known, that cubebs, and *sometimes* the balsam of copaiva, produces the effects I am about to describe; but students and junior practitioners may long remain ignorant of such occasional effects of these medicines, as I find, upon reference to works which are generally consulted, that no mention is made of them.

Captain P—, after trying various remedies for the cure of an obstinate yet trifling gleet, took, of his own accord, large doses of cubebs three times a day. On the fourth day from the commencement of the remedy, he complained of great restlessness, flushing of the face, sickness, and headach. On the fifth, he was covered with an eruption from head to foot, which was accompanied by a high degree of sympathetic fever. The peculiar appearances of the eruption I will attempt to describe in the next case, which was more severe, and attended by more strongly marked characters. Captain P— recovered in a few days, by the assistance of mild aperients, salines, and confinement to the house: he was, of course, desired to discontinue the cubebs. Upon a subsequent occasion, he again ventured to try the remedy, but he gave it up after taking one dose of it, in consequence of feeling the same general symptoms of restlessness, &c. which had preceded the former attack.

Madame T— had long laboured under severe leucorrhœa, and, from motives of delicacy, had neglected to consult any medical practitioner. She was recommended, by a friend, to try the effects of powder of cubebs, in doses of a small teaspoonful twice a day. After the first two doses, she felt feverish, with a tingling and heat over the whole surface of the body; she had rather severe headach, and a very disagreeable sensation of fulness in the eyes; the hands and feet felt hot and numbed. On the third day, a "flush of red" broke out over the whole of the body; and on the fourth, a decided eruption appeared. The cubebs was now discontinued, six doses having been taken. On the fifth day my attendance was required: her appearance was now peculiar, and rather formidable: her features were so completely changed as to leave no trace of their natural expression; the face was excessively swoln; eyes turgid and watery; lips puffed, dry, and shining; coryza; hands much swollen, and so stiff that the fingers could not be bent; respiration hurried and difficult; pulse small, and varying from 120 to 130; skin burning hot; intense thirst; tongue very white; constant nausea, and occasional vomiting. She was covered with an eruption from head to foot, which in some parts could not have been distinguished from Urticaria febrilis, while in others it had more the appearance of Lichen in its papular stage, but with much more intense inflammation around the base of the papulæ than is commonly seen in that disease. A fortnight elapsed before this lady completely recovered. The remedies employed consisted of purgatives, salines, and mild anodynes at bedtime.

Two or three other cases of the same kind, but milder in degree, have fallen under my own care, and several have been mentioned to me by other practitioners.

The following case was published in the Archives Générales for November 1831. A young man was admitted into La Pitié, under M. Velpeau, with gonorrhœa, which had existed for two months. After a few days, he was ordered a mixture containing cubebs, copaiva, and magnesia, in the proportions of two drachms of copaiva to four of cubebs. He took this mixture daily, and on the sixth day he was attacked with violent itching and burning over the head and neck. In the morning, his face was covered with dark-red patches, and in the course of a few hours this appearance of the skin, as well as the itching and sensation of burning, had extended over the chest and arms. The following day, the abdomen was attacked in the same manner. On the third day, the efflorescence that had at first appeared became pale, but the legs and feet now presented a similar appearance. The case might easily have been taken for measles, if the cutaneous symptoms only had been regarded, but there was none of the characteristic constitutional disturbance that attends that disease: the general health was, in fact, undisturbed. In the course of a few days the eruption entirely disappeared; and if any doubt had existed as to its cause, it was completely removed by the following circumstance: about three weeks after his recovery, the patient repeated a dose of the medicine, which he had accidentally kept; the eruption appeared on the following morning, and remained for two or three days.

*Upper Berkeley street, Portman square.*

